# Comparing Results of Five SARS-CoV-2 Antibody Assays Before and After the First Dose of ChAdOx1 nCoV-19 Vaccine among Health Care Workers

**DOI:** 10.1128/JCM.01105-21

**Published:** 2021-08-18

**Authors:** Seri Jeong, Nuri Lee, Su Kyung Lee, Eun-Jung Cho, Jungwon Hyun, Min-Jeong Park, Wonkeun Song, Eun Ju Jung, Heungjeong Woo, Yu Bin Seo, Jin Ju Park, Hyun Soo Kim

**Affiliations:** a Department of Laboratory Medicine, Kangnam Sacred Heart Hospital, Hallym University College of Medicine, Seoul, South Korea; b Department of Laboratory Medicine, Dongtan Sacred Heart Hospital, Hallym University College of Medicine, Hwaseong, South Korea; c Division of Infectious Diseases, Department of Internal Medicine, Dongtan Sacred Heart Hospital, Hallym University College of Medicine, Hwaseong, South Korea; d Division of Infectious Diseases, Department of Internal Medicine, Kangnam Sacred Heart Hospital, Hallym University College of Medicine, Seoul, South Korea; Cepheid

**Keywords:** SARS-CoV-2, antibody, assay, vaccine, titer, adverse reaction

## Abstract

Reliable results regarding serologic positivity for severe acute respiratory syndrome coronavirus 2 (SARS-CoV-2) antibody before and after AstraZeneca (AZ) vaccination are essential for estimating the efficacy of vaccination. We assessed positivity rates and associated factors using five SARS-CoV-2 antibody assays. A total of 228 paired serum samples (456 samples) were obtained from 228 participants. After baseline sampling, the second sampling was conducted between 11 and 28 days after the first dose of the AZ vaccine. Sera were tested using five SARS-CoV-2 antibody assays, including two surrogate virus neutralization tests. A questionnaire on the symptoms, severity, and duration of adverse reactions was completed by all participants. The overall positivity rates for SARS-CoV-2 antibody were 84.6% for the Roche assay, 92.5% for the Abbott assay, 75.4% for the Siemens assay, 90.7% for the SD Biosensor assay, and 66.2% for the GenScript assay after the first dose of the AZ vaccine. The positivity rates and antibody titers of sera obtained between 21 and 28 days were significantly higher than those obtained between 11 and 20 days in all five assays. More-severe adverse reactions and longer durations of adverse reactions were related to higher SARS-CoV-2 antibody levels. The agreements and correlations among the assays applied were substantial (к, 0.73 to 0.95) and strong (ρ, 0.83 to 0.91). A single dose of the AZ vaccine led to high positivity rates based on the five assays. Days after vaccination and adverse reactions could help estimate serologic conversion rates. The results should be interpreted cautiously considering the assays and cutoffs applied. Our findings could inform decisions regarding vaccination and laboratory settings and could thus contribute to the control of the spread of SARS-CoV-2 infection.

## INTRODUCTION

Coronavirus disease 2019 (COVID-19) is a viral respiratory syndrome caused by severe acute respiratory syndrome coronavirus 2 (SARS-CoV-2), which originated in Wuhan, Hubei Province, China, in December 2019. COVID-19 patients present with various symptoms, such as fever, cough, shortness of breath, and pneumonia, and the disease is characterized by rapid spread; the basal infection reproductive rate was approximately 3 (1–3). Currently, in South Korea, the AstraZeneca (AZ) vaccine (ChAdOx1 nCoV-19; AstraZeneca, Lund, Sweden) and the Pfizer-BioNTech (BNT162b2) vaccine (Pfizer, Inc., Philadelphia, PA) are administered to prevent the spread of COVID-19. To date, early data have been reported on the immunogenicity and safety of the vaccines (4–9), but factors related to vaccine effectiveness, such as the duration of the immune response and the rate of antibody production, remain unknown. In addition, insufficient clinical data on the association of antibody production with adverse reactions after vaccination, including allergic reactions, high fever, and chills, which are reported in several cases (10–12), have been published.

SARS-CoV-2 antibody testing aids in studying the immune responses of infected patients and identifying the precise serologic prevalence rate of infection in an affected area (13). SARS-CoV-2 antibody testing is currently performed using various measurement methods, including chemiluminescence immunoassays (CLIA) and enzyme-linked immunosorbent assays (ELISA) (14, 15). In performance evaluation, antibody testing shows a sensitivity of 90.1% to 97.4% and a specificity of 97.9% to 100%, depending on the assay reagents and equipment used (16). Quantitative evaluation of the SARS-CoV-2 antibody titer is the most intuitive and rapid approach to determining the effect of vaccination. However, performance evaluation for many methods based on CLIA and ELISA is still lacking for various clinical institutions. In particular, the performance of antibody testing using various CLIA methods in determining vaccine effectiveness has not been studied thus far.

Therefore, this study aimed to investigate the response rates of antibody production, including the production of neutralizing antibodies (NAbs), before and after vaccination among health care workers receiving the first dose of the AZ vaccine. We also investigated associated factors, including adverse reactions after vaccination, through a questionnaire. In addition, the results of five SARS-CoV-2 antibody assays after vaccination were compared in order to determine the best laboratory setting.

## MATERIALS AND METHODS

### Study population and sample collection.

A total of 228 health care workers from two university hospitals (Hallym University Dongtan Sacred Heart Hospital and Hallym University Kangnam Sacred Heart Hospital) were included in this study. The workers were older than 18 years and received the AZ vaccine between 4 and 12 March 2021. Serum samples were obtained from the participants to determine the presence of SARS-CoV-2 antibodies at baseline (*n* = 228). The second sampling was conducted between 11 and 28 days after the first dose to evaluate the serological response (*n* = 228). Initially, 234 participants were registered. Among them, three health care workers who did not receive vaccines were excluded. Three participants who received the Pfizer-BioNTech vaccine were also excluded. Finally, 456 serum samples (228 for baseline and 228 for serological response) from 228 participants were collected, aliquoted, and stored at −70°C until use.

This study was approved by the Institutional Review Board of Hallym University Dongtan Sacred Heart Hospital (HDT 2021-02-007) and the Institutional Review Board of Hallym University Kangnam Sacred Heart Hospital (HKS 2021-02-030-003). Informed consent was obtained from all participants.

### Questionnaire on adverse reactions after the first dose of AZ vaccination.

All participants received the questionnaire on adverse reactions after the first dose of AZ vaccination. The questionnaire comprised four questions regarding the presence, severity, and duration of adverse reactions after the first dose of the AZ vaccine and the use or nonuse of antipyretic drugs.

### SARS-CoV-2 antibody assays.

Sera were tested using the following five SARS-CoV-2 antibody assays: the Elecsys Anti-SARS-CoV-2 S total-antibody assay on the Cobas e801 platform (Roche Diagnostics, Mannheim, Germany), the SARS-CoV-2 IgG II Quant assay on the Alinity i platform (Abbott Laboratories Abbott Park, IL, USA), the SARS-CoV-2 IgG assay on the Atellica platform (Siemens, Munich, Germany), the STANDARD E SARS-CoV-2 nAb ELISA kit (SD Biosensor, Suwon, Korea), and the cPass SARS-CoV-2 neutralization antibody detection kit (GenScript, NJ, USA). The SD Biosensor ELISA and GenScript ELISA were performed using the Epoch microplate spectrophotometer (BioTek Instruments, Winooski, VT, USA) and ELx50 filter microplate washer (BioTek Instruments). The GenScript cPASS SARS-CoV-2 neutralization antibody detection kit and the SD Biosensor STANDARD E SARS-CoV-2 nAb ELISA kit are surrogate virus neutralization tests that can detect neutralizing antibodies that can block the interaction between the receptor-binding domain (RBD) in reagents and ACE2 coating the ELISA plate. The SD Biosensor STANDARD E SARS-CoV-2 nAb ELISA kit is composed of the V1 and V2 assays: the V1 assay uses the V1 enzyme conjugate (the receptor-binding domain of the Wuhan/UK variant conjugated to horseradish peroxidase), and the V2 assay uses the V2 enzyme conjugate (the receptor-binding domain of the South Africa/Brazil variant conjugated to horseradish peroxidase). Therefore, this kit could detect SARS-CoV-2 antibodies against the UK, South Africa, and Brazil variants as well as the original SARS-CoV-2. At least one positive result in the V1 assay or V2 assay was interpreted as a positive result for SARS-CoV-2 neutralizing antibody using the SD Biosensor assay.

The principle, instrument, detecting antibody, reagents used, sample volume, cutoff value, and time to the first result of each assay are listed in Table 1. All procedures were performed according to the manufacturer’s instructions. Most assays were performed at Hallym University Dongtan Sacred Heart Hospital by one laboratory technician and one scientific researcher, but the SARS-CoV-2 IgG assay on the Atellica platform was performed at Hallym University Kangnam Sacred Heart Hospital by another laboratory technician. The coded samples were tested in a single-blinded manner with no prior information on the samples.

**TABLE 1:** Characteristics of the five SARS-CoV-2 antibody assays^*a*^

Variable	Details for the following assay:				
	Roche	Abbott	Siemens	SD Biosensor	GenScript
Product name	Elecsys Anti-SARS-CoV-2 S	SARS-CoV-2 IgG II Quant	SARS-CoV-2 IgG (sCOVG)	STANDARD E SARS-CoV-2 nAb ELISA	cPass SARS-CoV-2 neutralization antibody detection kit
Analyzer	Elecsys Cobas e801	Alinity i	Atellica IM	ELISA	ELISA
Principle	ECLIA	CMIA	CLIA	ELISA, sVNT	ELISA, sVNT
Target antibody	Anti-RBD, total	Anti-RBD, IgG	Anti-RBD, IgG	RBD-binding NAb	RBD-binding NAb
Antigen reagent(s) used	Biotinylated RBD	RBD-coated microparticle	RBD-coated microparticle	HRP-labeled RBD	HRP-labeled RBD
	RBD labeled with a ruthenium complex		Acridinium ester-labeled RBD	ACE2 coating an ELISA plate	ACE2 coating an ELISA plate
Sample type	Serum, plasma	Serum, plasma	Serum, plasma	Serum, plasma	Serum, plasma
Sample vol	12 μl	25 μl	40 μl	60 μl × 2	10 μl
Measuring range	0.4–250 (U/ml)	21–40,000 (AU/ml)	0.5–150 (index)	0–100 (PI value)	0–100 (% signal inhibition)
Cutoff value (unit)	0.8 (U/ml)	50 (AU/ml)	1.0 (index)	30 (PI value)	30 (% signal inhibition)
Time to first result (min)	18	29	15	95	80

a ECLIA, electrochemiluminescence immunoassay; CMIA, chemiluminescence microparticle immunoassay; CLIA, chemiluminescence immunoassay; ELISA, enzyme-linked immunosorbent assay; sVNT, surrogate virus neutralization test; NAb, neutralizing antibody; HRP, horseradish peroxidase; RBD, receptor-binding domain; AU, arbitrary units; PI, percent inhibition.

### Statistical analysis.

Statistical analysis was performed using MedCalc software, version 19.8 (MedCalc Software Ltd., Ostend, Belgium), and Analyse-it Method Evaluation Edition software, version 2.26 (Analyse-it Software Ltd., Leeds, UK). Positivity was calculated according to subgroups based on participants’ characteristics. Comparisons of nominal and continuous variables were assessed using the chi-square test, Mann-Whitney U test, and Kruskal-Wallis test. Positive, negative, and total agreements between assays were evaluated using Cohen’s kappa (к) statistics, with the categories of poor (below 0.00), slight (0.00 to 0.20), fair (0.21 to 0.40), moderate (0.41 to 0.60), substantial (0.61 to 0.80), and almost perfect (0.81 to 1.00). Spearman’s rank correlation coefficients for the correlations among the five SARS-CoV-2 antibody assays were calculated and expressed as correlation graphs. They were interpreted as negligible (<0.1), weak (0.1 to 0.39), moderate (0.40 to 0.69), strong (0.70 to 0.89), or very strong (≥0.9).

### Data availability.

The data set for this article has been deposited at https://dataverse.harvard.edu/ (17).

## RESULTS

### Characteristics of participants and samples.

A total of 456 serum samples from 228 participants were collected. The demographic data of the vaccinated participants and serological positivity rates are presented in Table 2. The median age of the participants was 33.5 years (1st to 3rd quartile range, 27.0 to 44.0 years). Of the 228 samples obtained after the first vaccination, 179 (78.5%) were sampled before 3 weeks (11 to 20 days) and 49 (21.5%) were collected after 3 weeks (21 to 28 days). In our cohort, nurses accounted for 67.5%, medical laboratory technicians for 25.4%, and doctors for 6.1%. Most participants experienced mild adverse reactions after vaccination (*n* = 152 [66.7%]). Among individuals with adverse reactions, most experienced the reactions for 2 to 3 days (*n* = 141 [64.1%]). Antipyretics were administered to 89.9% of participants. All participants were given two Tylenol tablets, and the medical staff were instructed to take the tablets if adverse reactions occurred at the vaccine administration site. However, many participants took them prophylactically before the adverse reactions occurred.

**TABLE 2:** Positivity rates of SARS-CoV-2 antibody assays according to the characteristics of the participants^*a*^

Characteristic (*n*)	Roche assay		Abbott assay		Siemens assay		SD Biosensor assay		GenScript assay	
	Positivity rate^*a*^	*P* value^*b*^	Positivity rate	*P* value	Positivity rate	*P* value	Positivity rate	*P* value	Positivity rate	*P* value
Before vaccination (228)	0 (0.0)		1 (0.4)		0 (0.0)		2 (0.9)		0 (0.0)	
After 1st vaccination (228)	193 (84.6)		211 (92.5)		172 (75.4)		206 (90.7)		151 (66.2)	
Sex		0.442		0.827		0.437		0.404		0.407
Male (36)	32 (88.9)		33 (91.7)		29 (80.6)		34 (94.4)		26 (72.2)	
Female (192)	161 (83.9)		178 (92.7)		143 (74.5)		172 (89.6)		125 (65.1)	
Age		0.686		0.586		0.685		0.925		0.828
21–30 (101)	85 (84.2)		95 (94.1)		76 (75.2)		91 (90.1)		70 (69.3)	
31–40 (50)	43 (86.0)		44 (88.0)		39 (78.0)		45 (90.0)		31 (62.0)	
41–50 (46)	37 (80.4)		43 (93.5)		32 (69.6)		41 (89.1)		30 (65.2)	
51–60 (31)	28 (90.3)		29 (93.5)		25 (80.6)		29 (93.5)		20 (64.5)	
Occupation		0.143		0.508		0.154		0.284		0.160
Doctor (14)	14 (100.0)		14 (100.0)		13 (92.9)		14 (100.0)		13 (92.9)	
Nurse (154)	127 (82.5)		140 (90.9)		110 (71.4)		136 (88.3)		98 (63.6)	
Laboratory technician (58)	51 (87.9)		55 (94.8)		47 (81.0)		54 (93.1)		39 (67.2)	
Others (2)	1 (50.0)		2 (100.0)		2 (100.0)		2 (100.0)		1 (50.0)	
Days after 1st vaccination		**0.004**		**0.025**		**0.008**		**0.049**		**0.010**
11–20 (179)	145 (81.0)		162 (90.5)		128 (71.5)		158 (88.3)		111 (62.0)	
21–28 (49)	48 (98.0)		49 (100.0)		44 (89.8)		48 (98.0)		40 (81.6)	
Adverse reactions after 1st vaccination		0.809		0.479		**<0.001**		0.510		**0.018**
Absent (8)	7 (87.5)		7 (87.5)		2 (25.0)		7 (87.5)		3 (37.5)	
Mild (152)	127 (83.6)		139 (91.4)		111 (73.0)		135 (88.8)		95 (62.5)	
Severe (68)	59 (86.8)		65 (95.6)		59 (86.8)		64 (94.1)		53 (77.9)	
Duration (days) of adverse reactions		**0.011**		**<0.001**		**0.002**		**<0.001**		0.191
<1 (59)	43 (72.9)		48 (81.4)		36 (61.0)		46 (78.0)		35 (59.3)	
2–3 (141)	124 (87.9)		136 (96.5)		116 (82.3)		133 (94.3)		97 (68.8)	
>4 (20)	19 (95.0)		20 (100.0)		18 (90.0)		20 (100.0)		16 (80.0)	
Antipyretics		0.350		0.550		0.858		0.392		0.411
Taken (205)	172 (83.9)		189 (92.2)		155 (75.6)		184 (89.8)		134 (65.4)	
Not taken (23)	21 (91.3)		22 (95.7)		17 (73.9)		22 (95.7)		17 (73.9)	

*^a^*Positivity rates are expressed as number (percent).

*^b^P* values of <0.05 are given in boldface.

### Statistical analysis.

The positivity rates for SARS-CoV-2 antibody before vaccination among 228 health care workers were 0.0% for the Roche assay, 0.4% (1/228) for the Abbott assay, 0.0% for the Siemens assay, 0.9% (2/228) for the SD Biosensor assay, and 0.0% for the GenScript assay.

### Positivity rates by the five SARS-CoV-2 antibody assays after vaccination.

**FIG 1:**
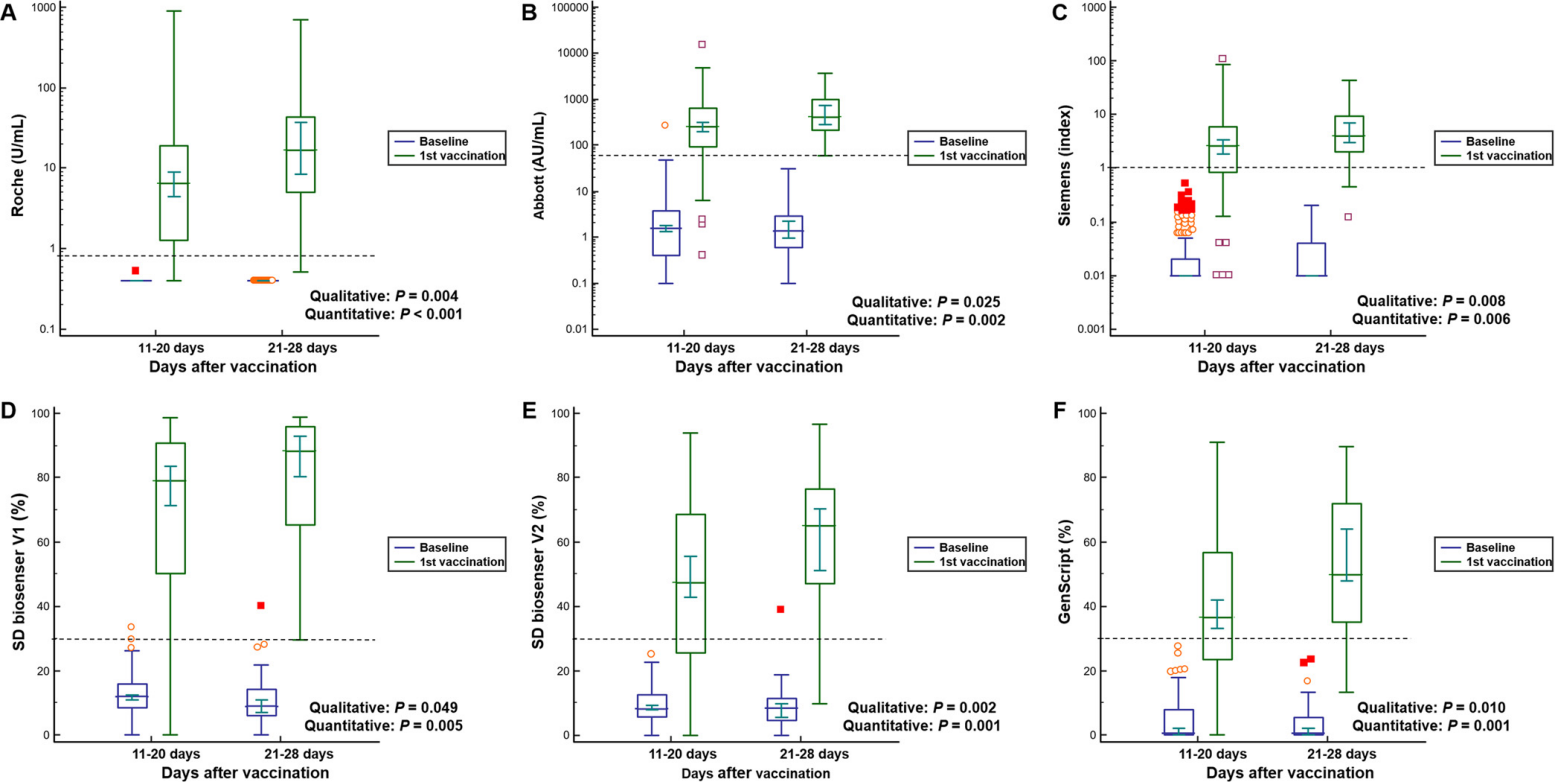
Serological responses to a single dose of the AstraZeneca vaccine according to the number of days after vaccination. (A) Roche; (B) Abbott; (C) Siemens; (D) SD Biosensor V1; (E) SD Biosensor V2; (F) GenScript. *P* values were calculated by the Mann-Whitney U test for the quantitative differences between 11 and 20 days and 21 to 28 days after the first vaccination. Chi-square tests were applied to the calculation of *P* values for the qualitative rates of positivity. The difference between baseline and the first vaccination was significant in all assays included (*P < *0.001). Dashed lines indicate the cutoff of each assay.

**FIG 2:**
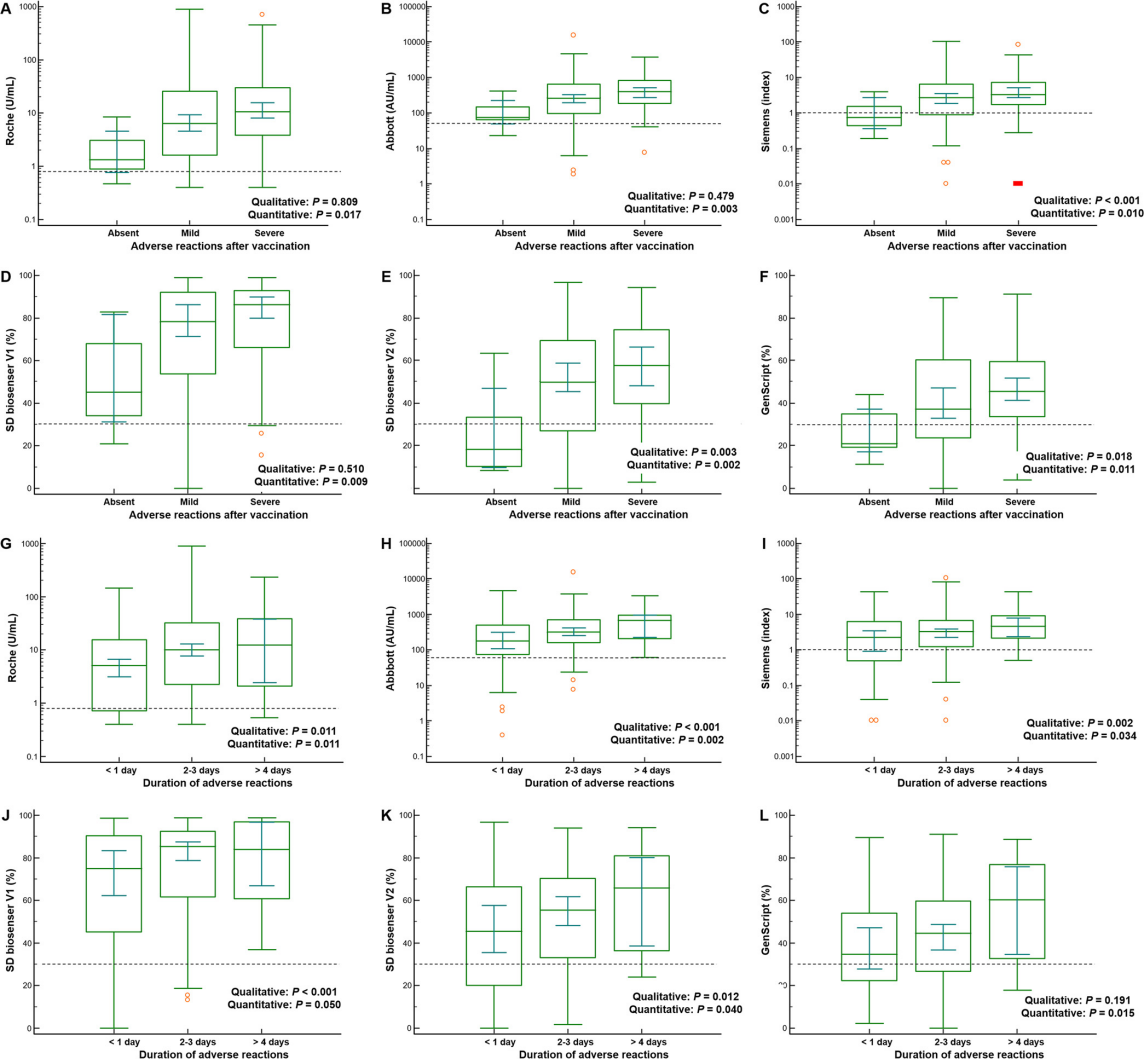
Comparison of serological responses to a single dose of the AstraZeneca vaccine according to adverse reactions. Shown are quantitative values related to the severity of adverse reactions for the Roche (A), Abbott (B), Siemens (C), SD Biosensor V1 (D), SD Biosensor V2 (E), and GenScript (F) assays and the quantitative assay values according to the duration of adverse reactions for the Roche (G), Abbott (H), Siemens (I), SD Biosensor V1 (J), SD Biosensor V2 (K), and GenScript (L) assays. *P* values were calculated by the Kruskal-Wallis test for the quantitative differences among <1 day, 2 to 3 days, and >4 days after the first vaccination. Chi-square tests were used for the calculation of *P* values for the qualitative rates of positivity. Dashed lines show the cutoff of each assay.

The overall positivity rates for SARS-CoV-2 antibody after the first dose of vaccine were 84.6% for the Roche assay, 92.5% for the Abbott assay, 75.4% for the Siemens assay, 90.7% for the SD Biosensor assay, and 66.2% for the GenScript assay (Table 2). The SD Biosensor V2 assay, targeting the antibody to the South Africa/Brazil variant of SARS-CoV-2, showed 72.2% positivity, whereas the SD Biosensor V1 assay, targeting the antibody to the original Wuhan SARS-CoV-2 and UK variant, revealed 90.7% positivity. The positivity rates of sera obtained between 21 and 28 days were significantly higher than those obtained between 11 and 20 days in all five assays. (98.0% [*P* = 0.004] for Roche, 100.0% [*P* = 0.025] for Abbott, 89.8% [*P* = 0.008] for Siemens, 98.0% [*P = *0.049] for SD Biosensor, and 81.6% [*P = *0.010] for GenScript). The absence of adverse reactions after vaccination showed significantly lower positivity rates in two assays than the presence of adverse reactions (25.0% [*P* < 0.001] for Siemens; 37.5% [*P* = 0.018] for GenScript). Adverse reactions lasting <1 day revealed lower positivity rates in four assays than those lasting 2 to 3 days or >4 days (72.9% [*P = *0.011] for Roche, 81.4% [*P* < 0.001] for Abbott, 61.0% [*P* = 0.002] for Siemens, and 78.0% [*P* < 0.001] for SD Biosensor). *P* values for qualitative rates of positivity are presented in Fig. 1 and 2. With regard to antipyretics, there were no significant differences between administrators and nonadministrators in terms of qualitative positivity (Table 2).

### Quantitative antibody values of the five SARS-CoV-2 antibody assays before and after the first vaccination.

Antibody levels before vaccination were available for 226 participants. The median values of the Abbott, SD Biosensor V1, and SD Biosensor V2 assays showing positivity in baseline samples were 1.5 AU/ml (1st to 3rd quartile range, 0.5 to 3.3 AU/ml), 11.2% (1st to 3rd quartile range, 7.5 to 15.4%), and 8.3% (1st to 3rd quartile range, 5.4 to 11.9%), respectively. After the first dose of the vaccine, the quantitative values of all five assays increased significantly (*P* < 0.001) (Fig. 1). The box-and-whisker plots shown in Fig. 1 and 2 depict the levels for variables showing significant differences in both qualitative and quantitative assessments. Quantitative levels at 21 to 28 days after vaccination were significantly higher than those at 11 to 20 days (medians, 16.8 versus 6.5 U/ml for the Roche assay, 417.0 versus 251.1 AU/ml for the Abbot assay, an index of 4.0 versus 2.6 for the Siemens assay, 88.3% versus 78.9% for the SD Biosensor V1 assay, 65.0% versus 47.3% for the SD Biosensor V2 assay, and 49.8% versus 36.6% for the GenScript assay) (Fig. 1). The presence and duration of adverse reactions also showed significant differences in the quantitative values of all five assays (Fig. 2). In terms of the quantitative antibody levels for the assays, male sex and the profession of doctor were related to higher values (see Table S1 in the supplemental material). Meanwhile, the intake of antipyretics was not associated with quantitative antibody values in any of the five assays (Table 2).

### Agreement and correlation of the five SARS-CoV-2 assays.

**FIG 3:**
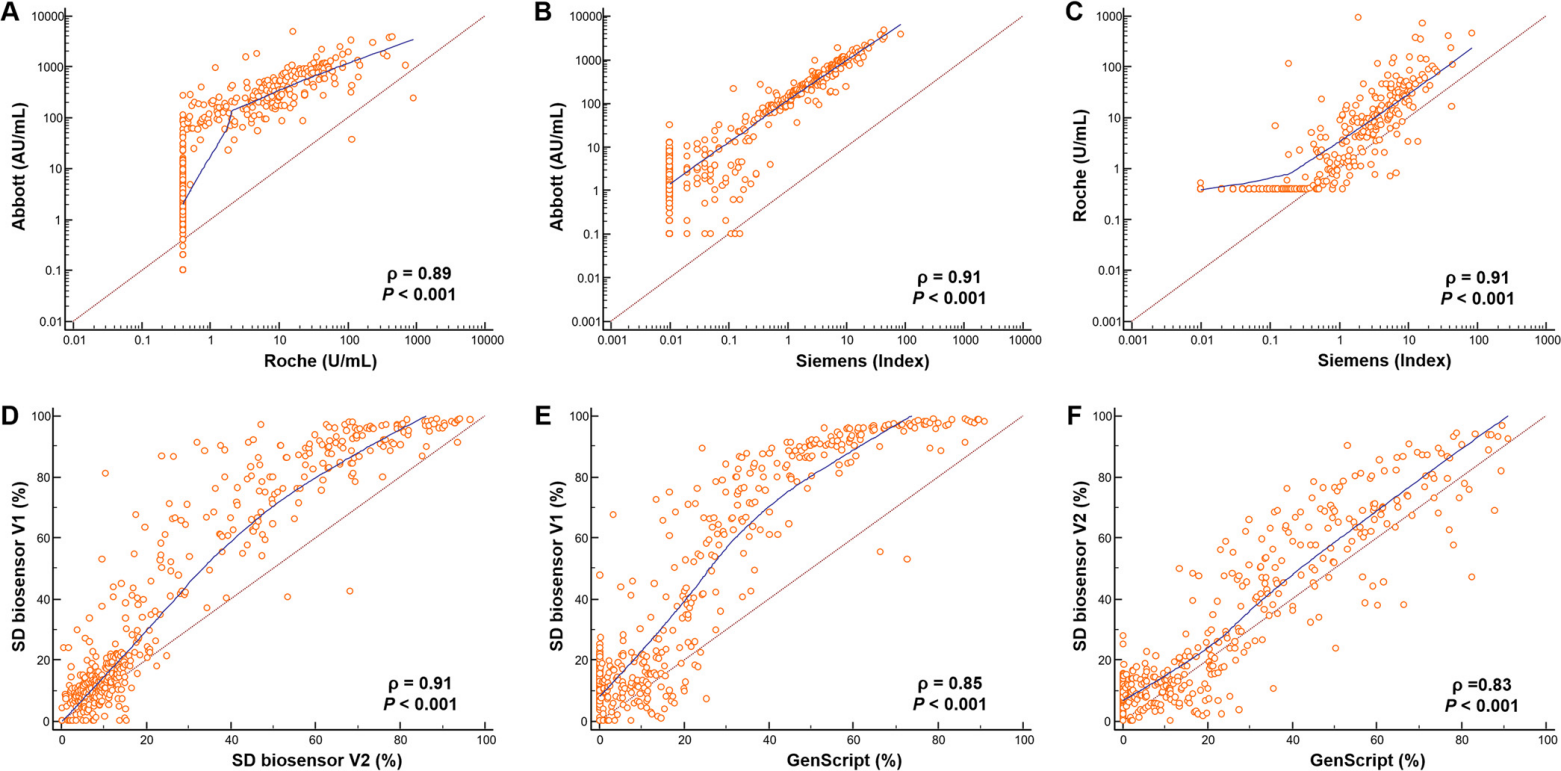
Correlation plots with ρ values of five SARS-CoV-2 antibody assays. (A) Abbott versus Roche; (B) Abbott versus Siemens; (C) Roche versus Siemens; (D) SD Biosensor V1 versus SD Biosensor V2; (E) SD Biosensor V1 versus GenScript; (F) SD Biosensor V2 versus GenScript.

The rates of agreement of the results among the five assays are summarized in Table 3. Total agreement rates ranged from 86.6% (95% confidence interval [CI], 83.1% to 89.6%) to 97.6% (95% CI, 95.7% to 98.8%). The Abbott and SD Biosensor assays showed the highest rate of agreement. There were no significant differences in agreement between nonneutralizing-antibody assays and neutralizing-antibody assays. There was substantial agreement among all the assays included based on kappa values ranging from 0.73 to 0.95. The Abbott and SD Biosensor assays showed the highest kappa value (0.95 [95% CI, 0.92 to 0.98]). Meanwhile, correlations among the assays were somewhat nonlinear (Fig. 3). Spearman’s correlation coefficients of rank correlation ranged from 0.83 to 0.91, showing strong correlation. The total number of discordant results among the five SARS-CoV-2 antibody assays was 90 (19.7%) out of 456 samples. Table 4 shows the numbers of samples (with median values) that presented positive or negative results in only one assay despite contrasting results in the other four assays. Among these, the most discrepant results were as follows: 5 (5.6%) samples showed positivity in the Abbott assay only, and 24 (26.7%) samples were positive by all assays except the GenScript assay.

**TABLE 3:** Rates of agreement between the five SARS-CoV-2 assays^*a*^

A/B	No. of samples with the following A/B pattern^*a*^:				Agreement (% [95% confidence interval])					Kappa value
	P/P	P/N	N/P	N/N	Positive, A to B	Negative, A to B	Positive, B to A	Negative, B to A	Total	
Roche/Abbott	190	3	22	239	89.6 (84.7–93.4)	98.8 (96.4–99.7)	98.4 (95.5–99.7)	91.6 (87.5–94.6)	94.5 (92.0–96.4)	0.89 (0.85–0.93)
Roche/Siemens	167	26	5	256	97.1 (93.3–99.1)	90.8 (86.8–93.9)	86.5 (80.9–91.0)	98.1 (95.6–99.4)	93.2 (91.4–95.3)	0.86 (0.81–0.91)
Roche/SD Biosensor	188	4	20	241	90.4 (85.5–94.0)	98.4 (95.9–99.6)	97.9 (94.8–99.4)	92.3 (88.4–95.3)	94.7 (92.2–96.6)	0.89 (0.85–0.93)
Roche/GenScript	148	45	3	258	98.0 (94.3–99.6)	85.1 (80.6–89.0)	76.7 (70.0–82.5)	98.9 (96.7–99.8)	89.4 (86.2–92.1)	0.78 (0.72–0.84)
Abbott/Siemens	171	41	1	241	99.4 (96.8–100.0)	85.5 (80.8–89.4)	80.7 (74.7–85.8)	99.6 (97.7–100.0)	90.7 (87.7–93.3)	0.81 (0.76–0.87)
Abbott/SD Biosensor	204	7	4	238	98.1 (95.2–99.5)	97.1 (94.2–98.8)	967 (93.3–98.7)	98.3 (95.8–99.5)	97.6 (95.7–98.8)	0.95 (0.92–0.98)
Abbott/GenScript	151	61	0	242	100.0 (97.6–100.0)	79.9 (74.9–84.2)	71.2 (64.6–77.2)	100.0 (98.5–100.0)	86.6 (83.1–89.6)	0.73 (0.66–0.79)
Siemens/SD Biosensor	170	1	38	244	81.7 (75.8–86.7)	99.6 (97.7–100.0)	99.4 (96.8–100.0)	86.5 (82.0–90.3)	91.4 (88.4–93.8)	0.82 (0.77–0.88)
Siemens/GenScript	144	28	7	275	95.4 (90.7–98.1)	90.8 (86.9–93.8)	83.7 (77.3–88.9)	97.5 (95.0–99.0)	92.3 (89.4–94.6)	0.83 (0.78–0.89)
SD Biosensor/GenScript	150	58	0	245	100.0 (97.6–100.0)	80.9 (76.0–85.1)	72.1 (65.5–78.1)	100.0 (98.5–100.0)	87.2 (83.8–90.1)	0.74 (0.68–0.80)

*^a^*N, negative; P, positive.

**TABLE 4:** Analysis of discrepant results among five SARS-CoV-2 antibody assays before and after the 1st dose of the AstraZeneca vaccine^*a*^

Category of discrepancy	No. (%) of samples	Median (range) antibody level by the following assay:				
		Roche (U/ml)	Abbott (AU/ml)	Siemens (index)	GenScript (%)	SD biosensor V1 (%)
Roche only positive	2 (2.2)	58.6 (2.3–115.0)	36.4 (36.0–36.8)	0.5 (0.0–1.0)	17.0 (9.9–24.1)	13.4 (4.6–22.2)
Abbott only positive	5 (5.6)	0.5 (0.0–1.0)	82.1 (61.0–267.3)	0.5 (0.4–0.8)	21.9 (14.0–27.6)	26.8 (24.3–29.7)
Siemens only positive	1 (1.1)	0.7	35.4	1.48	10.1	21.7
GenScript only positive	0	–	–	–	–	–
SD Biosensor only positive (V1 positive or V2 positive)	2 (2.2)	0.4 (0.4–0.4)	11.2 (0.6–21.7)	0.2 (0.1–0.3)	13.6 (7.5–19.7)	38.2 (33.5–41.1)
Roche only negative	1 (1.1)	0.7	182.1	5.54	32.3	79.9
Abbott only negative	0	–	–	–	–	–
Siemens only negative	5 (5.6)	1.7 (0.9–23.0)	86.3 (76.0–150.2)	0.7 (0.5–1.0)	35.6 (31.6–60.3)	68.3 (62.6–90.2)
GenScript only negative	24 (26.7)	4.5 (1.1–114.0)	193.1 (107.0–950.8)	1.7 (1.1–5.1)	25.8 (13.0–30.0)	65.0 (41.6–89.3)
SD Biosensor only negative	0	–	–	–	–	–
Others	50 (55.6)					
Total	90 (100)	1.5 (0.4–143.0)	115.1 (0.6–1347.6)	0.9 (0.0–15.5)	24.2 (0.1–72.9)	54.2 (4.6–90.2)

a Cutoff values are 0.8 U/ml for the Roche assay, 50 AU/ml for the Abbott assay, an index of 1.0 for the Siemens assay, 30% for the GenScript assay, and 30% for the SD Biosensor V1 assay.

## DISCUSSION

In this study, we investigated antibody responses to a single dose of the AZ vaccine using five SARS-CoV-2 assays consisting of three binding antibody (nonneutralizing antibody) assays and two surrogate virus neutralizing antibody assays. Our results showed 66.2% to 92.5% seroconversion rates, which differed from one assay to another. The number of days after vaccination, the presence of adverse reactions, and the duration of adverse reactions were related to the positivity rates. The agreements and correlations among the five assays were substantial and strong.

Our results showed an increase in the antispike antibody responses after the first dose of the AZ vaccine, in accordance with the result of a previous report on the safety and immunogenicity of the ChAdOx1 nCoV-19 vaccine, in which a single vaccination exhibited a significant increase in antispike antibody responses, peaking by day 28 in 543 subjects (18). With regard to the neutralizing ability of the antibody, the study showed that the response rates after ChAdOx1 nCoV-19 vaccination were 91% (32/35) and 100% (35/35) using a microneutralization assay and a plaque reduction neutralization test, respectively (18). Our results obtained through surrogate virus neutralization tests revealed 90.7% (206/228) positivity in the SD Biosensor assay and 66.2% (151/228) positivity in the GenScript assay. The positivity of the SD Biosensor assay in our study was similar to that of assays used in a previous study (18). However, there are studies demonstrating an effectiveness of 67% (19) or a pooled efficacy of 76% (20) despite the high seroconversion rate (more than 90%) after the first AZ vaccination. Furthermore, the GenScript assay showed a level of accuracy for qualitative delineation between individuals with positive and negative results similar to or higher than those of eight SARS-CoV-2 IgG serology and two live-cell neutralization tests (21). Considering these reports, the positivity of the GenScript assay in our study may be associated with actual protection against SARS-CoV-2 infection.

According to a previous study performed in Oxford University Hospitals, the positivity rates of SARS-CoV-2 antispike antibody responses to the first dose of the AZ vaccine in participants with no prior evidence of infection were 94% in 15 to 21 days and 97% in 22 to 28 days (22). In addition, Singh et al. (23) demonstrated that antibody titers between 21 and 28 days after the first dose were higher than those at other times. An interim analysis of four randomized controlled trials showed two cases of hospitalization because of SARS-CoV-2 infection before 21 days after the first dose, whereas no hospitalized individual was found after 21 days (8). Similarly to previous reports, our study results revealed higher positivity rates between 21 and 28 days than between 11 and 20 days in all SARS-CoV-2 antibody assays included. Two participants who had negative results for SARS-CoV-2 antibody assays when sampled between 14 and 20 days showed positive conversions in samples collected between 42 and 47 days, suggesting the importance of the number of days after vaccination (data not shown).

Local and systemic adverse reactions, including injection site pain, chills, muscle ache, and headache, have also been reported (18). Many reactions were resolved by the use of paracetamol and were less common in older adults (18, 24). However, there has been a lack of reports on the association of adverse reactions with serologic responses. To investigate this possible association, we collected data on the symptoms, severity, and duration of adverse reactions and the use of antipyretics through a questionnaire. The presence and the duration of adverse reactions were significantly related to the positivity rate in qualitative and quantitative analyses in our study. Therefore, these variables may be useful as subjective surrogate parameters to estimate the production of anti-SARS-CoV-2 antibodies.

Older age was reported to be associated with lower rates of seroconversion (22, 23). However, in our study, age was not related to positivity rates of SARS-CoV-2 antibodies in any of the five assays, a finding consistent with the results of a previous study demonstrating similar levels of immunogenicity across all age groups (24). Study population characteristics such as the number of subjects included, distribution, and ethnicity might influence these results.

With regard to the immunogenicity of the AZ vaccine against variants of SARS-CoV-2, there is a discordance of results among reported studies. A study conducted in South Africa revealed that the B.1.351 variant exhibited increased resistance as determined by neutralization assays (25). Meanwhile, another study suggests that a single dose of vaccine based on the original sequence might induce a significant increase in antibodies cross-reactive with variants such as B.1.351 and B.1.1.7 (26). According to another study conducted at the University of Oxford (19), two vaccine doses provided >85% protection against SARS-CoV-2 infection in health care workers, and this included protection against the B.1.1.7 variant. The SD Biosensor V2 assay is designed to detect antibodies to the South Africa/Brazil variant of SARS-CoV-2. However, no samples in this study showed SD Biosensor V1-negative and V2-positive results, which would suggest antibodies to the South Africa/Brazil SARS-CoV-2 variant. All SD Biosensor V2-positive samples (165/165) showed V1-positive results, whereas 43 of 208 V1-positive samples (20.7%) showed V2-negative results.

The five representative immunoassays included in this study showed substantial agreement and strong correlation with each other, with kappa values of 0.73 to 0.95 and Spearman’s correlation coefficients of 0.83 to 0.91. We analyzed the results showing inconsistency, where 90 samples showed discrepancies among the five SARS-CoV-2 antibody assays. Most inconsistencies were found in specimens classified as positive or negative with a quantitative value near the cutoff value at the time of seroconversion. These discrepancies affected the positivity rates, resulting in 66.2% for the GenScript assay and 92.5% for the Abbott assay. If the previous 20% cutoff value for the GenScript assay were used rather than the current cutoff of 30%, then its positivity rate would increase (27, 28). The assays and cutoff values applied are important factors for analyses. Laboratories can adjust their cutoff values according to the intended purpose. Other points to consider in interpreting the results of SARS-CoV-2 antibody assays are the types of antibodies detected and the reagent antigens used. The Elecsys assay measures total antibody, whereas the Abbott and Siemens assays measure IgG only. Total antibody contains not only IgG but also IgM and IgA, which can affect antibody positivity according to the days after vaccination.

One limitation of this study was the single value of short-term antibody measurement after the first vaccination. This preliminary report could not predict the final efficacy of vaccination. Serial evaluation of serological responses with longer periods after the completion of the second dose may be an ideal reflection of the effect of vaccination. In addition, focusing on defined health care workers can be both a strength and a weakness. Additional assessment in children and older individuals aged >60 years is necessary.

In conclusion, a single dose of the AZ vaccine induced high positivity based on five representative SARS-CoV-2 antibody assays. The number of days after vaccination, the presence of adverse events, and the duration of adverse reactions were associated with higher serologic conversion rates and increased antibody titers. The agreements and correlations among the assays applied were substantial and strong, but the results should be interpreted with caution considering the characteristics of the adopted assay and the cutoff values. To the best of our knowledge, this is the first report to provide reliable serological results after AZ vaccination based on five representative SARS-CoV-2 antibody assays, including neutralization antibody assays. In addition, this study includes information about serological responses of the East Asian population. The results of our evaluation should facilitate precise decision making for vaccination and contribute to the control of the spread of SARS-CoV-2 infection.

## References

[1] George PM, Wells AU, Jenkins RG. 2020. Pulmonary fibrosis and COVID-19: the potential role for antifibrotic therapy. Lancet Respir Med 8:807–815. 10.1016/S2213-2600(20)30225-3. 32422178 PMC7228727

[2] Kim YJ, Park H, Choi YY, Kim YK, Yoon Y, Kim KR, Choi EH. 2020. Defining association between COVID-19 and the multisystem inflammatory syndrome in children through the pandemic. J Korean Med Sci 35:e204. 10.3346/jkms.2020.35.e204. 32508068 PMC7279946

[3] Lee LY, Cazier JB, Angelis V, Arnold R, Bisht V, Campton NA, Chackathayil J, Cheng VW, Curley HM, Fittall MW, Freeman-Mills L, Gennatas S, Goel A, Hartley S, Hughes DJ, Kerr D, Lee AJ, Lee RJ, McGrath SE, Middleton CP, Murugaesu N, Newsom-Davis T, Okines AF, Olsson-Brown AC, Palles C, Pan Y, Pettengell R, Powles T, Protheroe EA, Purshouse K, Sharma-Oates A, Sivakumar S, Smith AJ, Starkey T, Turnbull CD, Várnai C, Yousaf N, Kerr R, Middleton G. 2020. COVID-19 mortality in patients with cancer on chemotherapy or other anticancer treatments: a prospective cohort study. Lancet 395:1919–1926. 10.1016/S0140-6736(20)31173-9. 32473682 PMC7255715

[4] Polack FP, Thomas SJ, Kitchin N, Absalon J, Gurtman A, Lockhart S, Perez JL, Pérez Marc G, Moreira ED, Zerbini C, Bailey R, Swanson KA, Roychoudhury S, Koury K, Li P, Kalina WV, Cooper D, Frenck RW, Jr, Hammitt LL, Türeci Ö, Nell H, Schaefer A, Ünal S, Tresnan DB, Mather S, Dormitzer PR, Şahin U, Jansen KU, Gruber WC, the C4591001 Clinical Trial Group. 2020. Safety and efficacy of the BNT162b2 mRNA Covid-19 vaccine. N Engl J Med 383:2603–2615. 10.1056/NEJMoa2034577. 33301246 PMC7745181

[5] Teijaro JR, Farber DL. 2021. COVID-19 vaccines: modes of immune activation and future challenges. Nat Rev Immunol 21:195–197. 10.1038/s41577-021-00526-x. 33674759 PMC7934118

[6] Zhu FC, Guan XH, Li YH, Huang JY, Jiang T, Hou LH, Li JX, Yang BF, Wang L, Wang WJ, Wu SP, Wang Z, Wu XH, Xu JJ, Zhang Z, Jia SY, Wang BS, Hu Y, Liu JJ, Zhang J, Qian XA, Li Q, Pan HX, Jiang HD, Deng P, Gou JB, Wang XW, Wang XH, Chen W. 2020. Immunogenicity and safety of a recombinant adenovirus type-5-vectored COVID-19 vaccine in healthy adults aged 18 years or older: a randomised, double-blind, placebo-controlled, phase 2 trial. Lancet 396:479–488. 10.1016/S0140-6736(20)31605-6. 32702299 PMC7836858

[7] Logunov DY, Dolzhikova IV, Zubkova OV, Tukhvatulin AI, Shcheblyakov DV, Dzharullaeva AS, Grousova DM, Erokhova AS, Kovyrshina AV, Botikov AG, Izhaeva FM, Popova O, Ozharovskaya TA, Esmagambetov IB, Favorskaya IA, Zrelkin DI, Voronina DV, Shcherbinin DN, Semikhin AS, Simakova YV, Tokarskaya EA, Lubenets NL, Egorova DA, Shmarov MM, Nikitenko NA, Morozova LF, Smolyarchuk EA, Kryukov EV, Babira VF, Borisevich SV, Naroditsky BS, Gintsburg AL. 2020. Safety and immunogenicity of an rAd26 and rAd5 vector-based heterologous prime-boost COVID-19 vaccine in two formulations: two open, non-randomised phase 1/2 studies from Russia. Lancet 396:887–897. 10.1016/S0140-6736(20)31866-3. 32896291 PMC7471804

[8] Voysey M, Clemens SAC, Madhi SA, Weckx LY, Folegatti PM, Aley PK, Angus B, Baillie VL, Barnabas SL, Bhorat QE, Bibi S, Briner C, Cicconi P, Collins AM, Colin-Jones R, Cutland CL, Darton TC, Dheda K, Duncan CJA, Emary KRW, Ewer KJ, Fairlie L, Faust SN, Feng S, Ferreira DM, Finn A, Goodman AL, Green CM, Green CA, Heath PT, Hill C, Hill H, Hirsch I, Hodgson SHC, Izu A, Jackson S, Jenkin D, Joe CCD, Kerridge S, Koen A, Kwatra G, Lazarus R, Lawrie AM, Lelliott A, Libri V, Lillie PJ, Mallory R, Mendes AVA, Milan EP, Minassian AM, Oxford COVID Vaccine Trial Group, et al. 2021. Safety and efficacy of the ChAdOx1 nCoV-19 vaccine (AZD1222) against SARS-CoV-2: an interim analysis of four randomised controlled trials in Brazil, South Africa, and the UK. Lancet 397:99–111. 10.1016/S0140-6736(20)32661-1. 33306989 PMC7723445

[9] Emary KRW, Golubchik T, Aley PK, Ariani CV, Angus B, Bibi S, Blane B, Bonsall D, Cicconi P, Charlton S, Clutterbuck EA, Collins AM, Cox T, Darton TC, Dold C, Douglas AD, Duncan CJA, Ewer KJ, Flaxman AL, Faust SN, Ferreira DM, Feng S, Finn A, Folegatti PM, Fuskova M, Galiza E, Goodman AL, Green CM, Green CA, Greenland M, Hallis B, Heath PT, Hay J, Hill HC, Jenkin D, Kerridge S, Lazarus R, Libri V, Lillie PJ, Ludden C, Marchevsky NG, Minassian AM, McGregor AC, Mujadidi YF, Phillips DJ, Plested E, Pollock KM, Robinson H, Smith A, Song R, Oxford COVID-19 Vaccine Trial Group, et al. 2021. Efficacy of ChAdOx1 nCoV-19 (AZD1222) vaccine against SARS-CoV-2 variant of concern 202012/01 (B.1.1.7): an exploratory analysis of a randomised controlled trial. Lancet 397:1351–1362. 10.1016/S0140-6736(21)00628-0. 33798499 PMC8009612

[10] Shimabukuro T, Nair N. 2021. Allergic reactions including anaphylaxis after receipt of the first dose of Pfizer-BioNTech COVID-19 vaccine. JAMA 325:780–781. 10.1001/jama.2021.0600. 33475702 PMC8892260

[11] Smadja DM, Yue QY, Chocron R, Sanchez O, Lillo-Le Louet A. 2021. Vaccination against COVID-19: insight from arterial and venous thrombosis occurrence using data from VigiBase. Eur Respir J 58:2100956. 10.1183/13993003.00956-2021. 33863748 PMC8051185

[12] Blauenfeldt RA, Kristensen SR, Ernstsen SL, Kristensen CCH, Simonsen CZ, Hvas AM. 2021. Thrombocytopenia with acute ischemic stroke and bleeding in a patient newly vaccinated with an adenoviral vector-based COVID-19 vaccine. J Thromb Haemost 19:1771–1775. 10.1111/jth.15347. 33877737 PMC8250306

[13] Noh JY, Seo YB, Yoon JG, Seong H, Hyun H, Lee J, Lee N, Jung S, Park MJ, Song W, Yoon J, Lim CS, Ryou J, Lee JY, Kim SS, Cheong HJ, Kim WJ, Yoon SY, Song JY. 2020. Seroprevalence of anti-SARS-CoV-2 antibodies among outpatients in southwestern Seoul, Korea. J Korean Med Sci 35:e311. 10.3346/jkms.2020.35.e311. 32830472 PMC7445312

[14] Higgins V, Fabros A, Kulasingam V. 2021. Quantitative measurement of anti-SARS-CoV-2 antibodies: analytical and clinical evaluation. J Clin Microbiol 59:e03149-20. 10.1128/JCM.03149-20. 33483360 PMC8092751

[15] Verkerke H, Horwath M, Saeedi B, Boyer D, Allen JW, Owens J, Arthur CM, Nakahara H, Rha J, Patel K, Wu SC, Paul A, Yasin N, Wang J, Shin S, Brown D, Normile K, Cole L, Meyers M, Lin H, Woods E, Isaac J, Broder K, Wade J, Kauffman RC, Patel R, Josephson CD, Reynolds S, Sherman M, Wrammert J, Alter D, Guarner J, Roback JD, Neish A, Stowell SR. 2021. Comparison of antibody class-specific SARS-CoV-2 serologies for the diagnosis of acute COVID-19. J Clin Microbiol 59:e02026-20. 10.1128/JCM.02026-20. 33468605 PMC8092741

[16] Chen SY, Lee YL, Lin YC, Lee NY, Liao CH, Hung YP, Lu MC, Wu JL, Tseng WP, Lin CH, Chung MY, Kang CM, Lee YF, Lee TF, Cheng CY, Chen CP, Huang CH, Liu CE, Cheng SH, Ko WC, Hsueh PR, Chen SC. 2020. Multicenter evaluation of two chemiluminescence and three lateral flow immunoassays for the diagnosis of COVID-19 and assessment of antibody dynamic responses to SARS-CoV-2 in Taiwan. Emerg Microbes Infect 9:2157–2168. 10.1080/22221751.2020.1825016. 32940547 PMC7580576

[17] Jeong S, Lee N, Kim HS. 2021. Data set of serological responses of 456 samples from 228 participants using five SARS-CoV-2 antibody assays. Deposited in https://dataverse.harvard.edu/.

[18] Folegatti PM, Ewer KJ, Aley PK, Angus B, Becker S, Belij-Rammerstorfer S, Bellamy D, Bibi S, Bittaye M, Clutterbuck EA, Dold C, Faust SN, Finn A, Flaxman AL, Hallis B, Heath P, Jenkin D, Lazarus R, Makinson R, Minassian AM, Pollock KM, Ramasamy M, Robinson H, Snape M, Tarrant R, Voysey M, Green C, Douglas AD, Hill AVS, Lambe T, Gilbert SC, Pollard AJ, Aboagye J, Adams K, Ali A, Allen E, Allison JL, Anslow R, Arbe-Barnes EH, Babbage G, Baillie K, Baker M, Baker N, Baker P, Baleanu I, Ballaminut J, Barnes E, Barrett J, Bates L, Batten A, et al. 2020. Safety and immunogenicity of the ChAdOx1 nCoV-19 vaccine against SARS-CoV-2: a preliminary report of a phase 1/2, single-blind, randomised controlled trial. Lancet 396:467–478. 10.1016/S0140-6736(20)31604-4. 32702298 PMC7445431

[19] Lumley SF, Rodger G, Constantinides B, Sanderson N, Chau KK, Street TL, O’Donnell D, Howarth A, Hatch SB, Marsden BD, Cox S, James T, Warren F, Peck LJ, Ritter TG, de Toledo Z, Warren L, Axten D, Cornall RJ, Jones EY, Stuart DI, Screaton G, Ebner D, Hoosdally S, Chand M, Crook DW, O’Donnell A-M, Conlon CP, Pouwels KB, Walker AS, Peto TEA, Hopkins S, Walker TM, Stoesser NE, Matthews PC, Jeffery K, Eyre DW. 2021. An observational cohort study on the incidence of SARS-CoV-2 infection and B.1.1.7 variant infection in healthcare workers by antibody and vaccination status. medRxiv. 10.1101/2021.03.09.21253218. PMC8994591 34216472

[20] Voysey M, Costa Clemens SA, Madhi SA, Weckx LY, Folegatti PM, Aley PK, Angus B, Baillie VL, Barnabas SL, Bhorat QE, Bibi S, Briner C, Cicconi P, Clutterbuck EA, Collins AM, Cutland CL, Darton TC, Dheda K, Dold C, Duncan CJA, Emary KRW, Ewer KJ, Flaxman A, Fairlie L, Faust SN, Feng S, Ferreira DM, Finn A, Galiza E, Goodman AL, Green CM, Green CA, Greenland M, Hill C, Hill HC, Hirsch I, Izu A, Jenkin D, Joe CCD, Kerridge S, Koen A, Kwatra G, Lazarus R, Libri V, Lillie PJ, Marchevsky NG, Marshall RP, Mendes AVA, Milan EP, Minassian AM, et al. 2021. Single-dose administration and the influence of the timing of the booster dose on immunogenicity and efficacy of ChAdOx1 nCoV-19 (AZD1222) vaccine: a pooled analysis of four randomised trials. Lancet 397:881–891. 10.1016/S0140-6736(21)00432-3. 33617777 PMC7894131

[21] Taylor SC, Hurst B, Charlton CL, Bailey A, Kanji JN, McCarthy MK, Morrison TE, Huey L, Annen K, DomBourian MG, Knight V. 2021. A new SARS-CoV-2 dual-purpose serology test: highly accurate infection tracing and neutralizing antibody response detection. J Clin Microbiol 59:e02438-20. 10.1128/JCM.02438-20. 33500361 PMC8092720

[22] Eyre DW, Lumley SF, Wei J, Cox S, James T, Justice A, Jesuthasan G, O’Donnell D, Howarth A, Hatch SB, Marsden BD, Jones EY, Stuart DI, Ebner D, Hoosdally S, Crook DW, Peto TEA, Walker TM, Stoesser NE, Matthews PC, Pouwels KB, Walker AS, Jeffery K. 2021. Quantitative SARS-CoV-2 anti-spike responses to Pfizer-BioNTech and Oxford-AstraZeneca vaccines by previous infection status. medRxiv. 10.1101/2021.03.21.21254061. PMC8180449 34111577

[23] Singh AK, Phatak SR, Singh NK, Gupta A, Sharma A, Bhattacharjee K, Singh R. 2021. Antibody response after first-dose of ChAdOx1-nCOV (Covishield) and BBV-152 (Covaxin) amongst health care workers in India: preliminary results of cross-sectional coronavirus vaccine-induced antibody titre (COVAT) study. medRxiv. 10.1101/2021.04.07.21255078. PMC8461292 34600747

[24] Ramasamy MN, Minassian AM, Ewer KJ, Flaxman AL, Folegatti PM, Owens DR, Voysey M, Aley PK, Angus B, Babbage G, Belij-Rammerstorfer S, Berry L, Bibi S, Bittaye M, Cathie K, Chappell H, Charlton S, Cicconi P, Clutterbuck EA, Colin-Jones R, Dold C, Emary KRW, Fedosyuk S, Fuskova M, Gbesemete D, Green C, Hallis B, Hou MM, Jenkin D, Joe CCD, Kelly EJ, Kerridge S, Lawrie AM, Lelliott A, Lwin MN, Makinson R, Marchevsky NG, Mujadidi Y, Munro APS, Pacurar M, Plested E, Rand J, Rawlinson T, Rhead S, Robinson H, Ritchie AJ, Ross-Russell AL, Saich S, Singh N, Smith CC, Oxford COVID Vaccine Trial Group, et al. 2021. Safety and immunogenicity of ChAdOx1 nCoV-19 vaccine administered in a prime-boost regimen in young and old adults (COV002): a single-blind, randomised, controlled, phase 2/3 trial. Lancet 396:1979–1993. 10.1016/S0140-6736(20)32466-1. 33220855 PMC7674972

[25] Madhi SA, Baillie V, Cutland CL, Voysey M, Koen AL, Fairlie L, Padayachee SD, Dheda K, Barnabas SL, Bhorat QE, Briner C, Kwatra G, Ahmed K, Aley P, Bhikha S, Bhiman JN, Bhorat AE, Plessis J, Esmail A, Groenewald M, Horne E, Hwa S-H, Jose A, Lambe T, Laubscher M, Malahleha M, Masenya M, Masilela M, McKenzie S, Molapo K, Moultrie A, Oelofse S, Patel F, Pillay S, Rhead S, Rodel H, Rossouw L, Taoushanis C, Tegally H, Thombrayil A, Eck S, Wibmer CK, Durham NM, Kelly EJ, Villafana TL, Gilbert S, Pollard AJ, de Oliveira T, Moore PL, Sigal A, et al. 2021. Safety and efficacy of the ChAdOx1 nCoV-19 (AZD1222) Covid-19 vaccine against the B.1.351 variant in South Africa. medRxiv. 10.1101/2021.02.10.21251247. PMC7993410 33725432

[26] Jeewandara C, Kamaladasa A, Pushpakumara PD, Jayathilaka D, Abayrathna IS, Danasekara S, Guruge D, Ranasinghe T, Dayarathne S, Pathmanathan T, Somathilaka G, Madhusanka D, Tanussiya S, Tibutius TPJ, Kuruppu H, Wijesinghe A, Thashmi N, Milroy D, Nandasena A, Sanjeewani N, Wijayamuni R, Samaraweera S, Schimanski L, Tan TK, Dong T, Ogg GS, Townsend A, Malavige GN. 2021. Antibody and T-cell responses to a single dose of the AZD1222/Covishield vaccine in previously SARS-CoV-2 infected and naive health care workers in Sri Lanka. medRxiv. 10.1101/2021.04.09.21255194.

[27] Meyer B, Reimerink J, Torriani G, Brouwer F, Godeke GJ, Yerly S, Hoogerwerf M, Vuilleumier N, Kaiser L, Eckerle I, Reusken C. 2020. Validation and clinical evaluation of a SARS-CoV-2 surrogate virus neutralisation test (sVNT). Emerg Microbes Infect 9:2394–2403. 10.1080/22221751.2020.1835448. 33043818 PMC7605318

[28] Lee SM, Kim IS, Lim S, Lee SJ, Kim WJ, Shin KH, Moon SY, Chang CL. 2021. Comparison of serologic response of hospitalized COVID-19 patients using 8 immunoassays. J Korean Med Sci 36:e64. 10.3346/jkms.2021.36.e64. 33686810 PMC7940118

