# Supplementary material for: Comparing Results of Five SARS-CoV-2 Antibody Assays Before and After the First Dose of ChAdOx1 nCoV-19 Vaccine among Health Care Workers
Source: J Clin Microbiol. 2021 Aug 18;59(9):e01105-21. doi: 10.1128/JCM.01105-21 (PMC8373005; doi:10.1128/JCM.01105-21)
Supplement: Supplemental file 1 — Table S1. Download JCM.01105-21-s0001.pdf, PDF file, 0.04 MB [file jcm.01105-21-s0001.pdf]

Comparing Results of Five SARS-CoV-2 Antibody Assays Before and After the First Dose of ChAdOx1 nCoV-19 Vaccine among Health Care Workers

Seri Jeong,[a] Nuri Lee,[a] Su Kyung Lee,[b] Eun-Jung Cho,[b] Jungwon Hyun,[b] Min-Jeong Park,[a] Wonkeun Song,[a] Eun Ju Jung,[c] Heungjeong Woo,[c] Yu Bin Seo,[d] Jin Ju Park,[d] Hyun Soo Kim[b]#

Supplementary information

Supplemental Table 1. Quantitative median values of SARS-CoV-2 antibody assays according to the characteristics of participants

Supplemental Table 1 Quantitative median values of SARS-CoV-2 antibody assays according to the characteristics of participants[a]

Characteristics	Roche (U/mL)	*P*-value	Abbott (AU/mL)	*P*-value	Siemens (index)	*P*-value	SD biosensor V1 (%)	*P*-value	SD biosensor V2 (%)	*P*-value	GenScript (%)	*P*-value
Sex		0.070		0.081		**0.035**		0.184		**0.019**		0.274
Male (n = 36)	12.1		418.8		5.4		86.8		66.8		48.5	
Female (n = 192)	7.5		265.3		2.6		79.9		49.8		39.7	
Age		0.838		0.603		0.552		0.405		0.874		0.940
21-30 (n = 101)	9.0		306.0		3.1		79.9		52.2		40.3	
31-40 (n = 50)	6.4		329.2		3.8		85.5		53.7		42.9	
41-50 (n = 46)	6.8		225.1		2.1		71.5		47.1		36.7	
51-60 (n = 31)	8.1		280.8		2.7		82.3		55.2		43.9	
Occupation		0.130		**0.011**		**0.018**		**0.038**		**0.012**		**0.049**
Doctor (n = 14)	15.8		531.2		5.6		91.6		68.1		56.7	
Nurse (n = 154)	6.4		245.2		2.2		78.2		46.7		36.6	
Medical technician (n = 58)	9.3		418.8		3.6		83.7		58.6		43.7	
Others (n = 2)	16.0		360.5		4.0		74.4		55.7		37.3	
**Days after vaccination**		**< 0.001**		**0.002**		**0.006**		**0.005**		**0.001**		**0.001**
11-20 days (n = 179)	6.5		251.1		2.6		78.9		47.3		36.6	
21-28 days (n = 49)	16.8		417.0		4.0		88.3		65.0		49.8	
**Adverse reactions after vaccination**		**0.017**		**0.003**		**0.01**		**0.009**		**0.002**		**0.011**
Absent (n = 8)	1.4		76.6		0.7		45.1		18.2		21.0	
Mild (n = 152)	6.4		259.7		2.8		78.2		50.0		37.3	
Severe (n = 68)	10.6		394.8		3.4		86.2		57.7		45.6	
**Duration of adverse reactions**		**0.011**		**0.002**		**0.034**		**0.05**		**0.04**		**0.015**
< 1 day (n = 59)	5.1		182.1		2.2		74.8		45.5		34.8	
2-3 days (n = 141)	10.1		318.3		3.3		85.3		55.5		44.5	
> 4 days (n = 20)	12.6		687.6		4.7		84.1		65.8		60.3	
Antipyretics		0.875		0.353		0.705		0.594		0.094		0.836
Not taken (n = 23)	8.8		197.9		2.6		76.2		41.7		35.7	
Taken (n = 205)	7.6		284.2		3.1		81.6		52.7		40.8	
Total after vaccination (n = 228)	7.8		278.4		3.0		80.3		51.1		40.3	

[a]Data are expressed as medians.
